# Implications of the NDC80 complex on the tumor immune microenvironment and cell growth in pan-cancer

**DOI:** 10.7150/jca.96070

**Published:** 2024-10-14

**Authors:** Jia-Xing Wang, Teng-Yue Diao, Xue-Ling Yang, Ke Li, Jun-Le Yang, Xie-Qun Chen

**Affiliations:** 1 Institute of Hematology, Faculty of Life Sciences and Medicine, Northwest University, Xi'an 710069, Shaanxi, China.; 2 Hematology and Oncology Center, Affiliated Hospital of Northwest University and Xi'an No. 3 Hospital, Xi'an 710000, Shaanxi, China.; 3 Core Research Laboratory, The Second Affiliated Hospital, Xi'an Jiaotong University, Xi'an 710004, Shaanxi, China.; 4 Department of Nephrology, The Third Affiliated Hospital of Chongqing Medical University, Chongqing 401120, China.; 5 Department of Radiology, Affiliated Hospital of Northwest University and Xi'an No. 3 Hospital, Xi'an 710000, Shaanxi, China.

**Keywords:** NDC80 complex, Th2 cell infiltration, immune checkpoint, cell growth, TP53 mutation

## Abstract

**Background:** Previous evidence indicates that the NDC80 complex, a conserved spindle microtubule-binding component of the kinetochore, is overexpressed and associated with prognosis in certain cancer types. Herein, we assessed the expression and prognostic value of NDC80 complex components in pan-cancer and interrogated their potential functions in tumor context through multiple databases and software.

**Results:** Our findings showed that the expression of NDC80 complex components was aberrant across almost all cancer types and correlated positively with poor prognosis at the pan-cancer level. Furthermore, the expression levels of NDC80 complex components were positively associated with Th2 cell infiltration in the majority of cancer types. Additionally, higher expression of the NDC80 complex components was associated with increased immune checkpoint gene expression and TP53 mutation in specific cancer types. We also discovered that NDC80 complex components play pivotal roles in cell division, and the cell cycle within the tumor context. Moreover, knockdown of NDC80 significantly suppressed cell growth and inhibited the G1-S phase transition in two breast cancer cell lines.

**Conclusions:** Our study suggests that the NDC80 complex components could serve as reliable biomarkers for cancer detection and prognosis in pan-cancer, in addition to uncovering their role as cancer-promoting genes involved in Th2 cell infiltration, immune checkpoint, cell growth, and TP53 mutation.

## Introduction

Despite enormous efforts, cancer remains one of the most lethal diseases in the world. It is characterized by the uncontrolled growth of damaged cells with genetic and epigenetic alterations^1^. Such alterations lead to abnormal expression of genes that play a crucial role in cancer progression and patient outcome. Unfortunately, it is experimentally challenging and laborious to systematically decipher the diverse roles of the genes with altered expression in the tumor context. Bioinformatics provides a new platform for screening such abnormally expressed genes and further understanding their clinical significance and potential biological functions in cancer.

The NDC80 complex, which comprises four protein subunits, known as NDC80, NUF2, SPC24, and SPC25, is conserved from fungi to humans^2^. The NDC80 complex is identified to have essential and critical roles in microtubule binding within the outer kinetochore. Recent evidence suggests that the NDC80 complex components are significantly overexpressed and play significant roles in a variety of cancers. For instance, NDC80 is reported to be upregulated and predict poor prognosis in several human cancers, including lung adenocarcinoma (LUAD)^3^, pancreatic adenocarcinoma (PAAD)^4^, colon adenocarcinoma (COAD)^5^, osteosarcoma^6^, liver hepatocellular carcinoma (LIHC)^7^, and gastric cancer^8^. Knockdown of NDC80 in various human cancer cell lines suppresses proliferation and induces apoptosis *in vitro*^4^, ^5^, ^7^, ^8^. On the contrary, overexpression of NDC80 promotes cancer cell proliferation *in vivo* in a gastric cancer model^8^. NUF2, another NDC80 complex component is overexpressed and correlated with poor patient survival in multiple human cancers, such as LUAD^9^, LIHC^10^, breast cancer (BC)^11^, and PAAD^12^. NUF2 silencing could inhibit pancreatic cancer cell growth *in vitro* and *in vivo*^12^. SPC24 expression is significantly elevated in BC and is associated with poor patient prognosis^13^. SPC24 knockdown leads to attenuated cell growth and increased cell apoptosis in BC cells *in vitro*. Similar results have been found in thyroid adenocarcinoma (THCA)^14^, LIHC^15^, LUAD^16^, and osteosarcoma^17^. In addition, SPC25 has been identified to be significantly upregulated and is associated with poor survival in LIHC^18^, prostate adenocarcinoma (PRAD)^19^, BC^20^, LUAD, and lung squamous cell adenocarcinoma (LUSC)^21^. Knockdown of SPC25 impairs the cell growth of various human cancer cell lines *in vitro*^19^-^21^ and upregulation of SPC25 promotes hepatocellular cancer cell proliferation *in vitro* and tumor growth *in vivo* via accelerating the cell cycle^22^.

These observations suggest that NDC80 complex components can be used as biomarkers for cancer detection and prognosis in certain human cancer types. Further, they appear to be associated with increased cell proliferation. However, there remains a paucity of evidence on the relationship between NDC80 complex components and immune cell infiltration as well as immune checkpoint genes. Furthermore, the regulation of NDC80 complex components expression largely remains unknown. These rationales motivated the present pan-cancer study.

## Material and methods

### Data collection

The heatmap of NDC80 complex components expression across normal and tumor tissues in the TCGA/TARGET/GTEx database was obtained from the UCSC Xena web-based tool (https://xenabrowser.net/). This tool allows users to explore functional genomic data sets for correlations between genomic and phenotypic variables. The mRNA expression data used to investigate the expression of NDC80 complex components across the 33 cancer types was obtained from UCSC Xena based on the cancer genome atlas (TCGA) (https://cancergenome.nih.gov/) and Genotype-Tissue Expression (GTEX) (https://gtexportal.org/) databases. UALCAN database (https://ualcan.path.uab.edu/) was used to evaluate the correlation between NDC80 complex components expression and pathological stages in various cancers.

### Survival analysis

We used Kaplan Meier survival analyses based on the UCSC Xena web-based tool to analyze the prognostic value of the NDC80 complex components in pan-cancer. According to the median expression levels of the NDC80 complex components, patient samples were separated into two groups (high and low expression) to investigate the overall survival (OS), disease-specific survival (DSS), disease-free interval (DFI), and progression-free interval (PFI) with log-rank test statistics and *p*-values.

### Immune infiltration analysis

We downloaded the uniformly standardized pan-cancer dataset (TCGA Pan-Cancer (PANCAN, N=10535, G=60499)) from UCSC Xena, and extracted the expression data of the NDC80 complex components of each sample from it. Next, we analyzed the infiltration scores of 64 immune cells, and quantified the ImmmuneScore, StromaScore, and MicroenvironmentScore in each patient according to the corresponding genes expression using the R software package IOBR (version0.99.9, https://www.ncbi.nlm.nih.gov/pmc/articles/PMC8283787/) based on deconvo_xCell method. Eventually we obtained 67 categories of immune cell infiltration scores in 33 TCGA tumor types of 9555 tumor patient samples. We used the R software package - psych (version 2.1.6) to calculate Pearson's correlation coefficient between NDC80 complex genes and immune cell infiltration scores. In addition, we analyzed the correlation between NDC80 complex components and immune cell infiltration via the TIMER2.0 database (http://timer.cistrome.org/) to verify the above immune infiltration analysis by xCell algorithm.

### Association between NDC80 complex components and immune checkpoint

We analyzed the correlation of immune checkpoint with NDC80 complex components in various cancers via TCGA. The immune checkpoint genes expression was compared between two groups of patient samples that were divided based on the median expression levels of NDC80, NUF2, SPC24 and SPC25 respectively in BRCA, BLCA and LIHC. In addition, we evaluated the correlation between NDC80 complex components and immune checkpoint genes in pan-cancer through the TIMER2.0 database.

### Functional status of NDC80 complex components and enrichment analyses of NDC80 complex components related genes

1. studied the functional status of NDC80 complex components in various cancer types via CancerSEA at the single-cell level. The protocol for Gene Ontology (GO) function and Kyoto Encyclopedia of Genes and Genomes (KEGG) pathway enrichment analyses of NDC80 complex components related genes in the tumor context were as follow. First, we obtained the top 200 genes having similar expression patterns associated with NDC80, NUF2, SPC24 and SPC25 respectively in pan-cancer from the GEPIA2 database (http://gepia2.cancer-pku.cn). After the intersection analysis of these related genes by the InteractiVenn tool (http://www. interactivenn.net/index.html), we obtained 109 overlapping genes, which were further analyzed by GO and KEGG analyses using DAVID functional annotation clustering tool (https://david.ncifcrf.gov/summary.jsp).

### Protein-protein interaction (PPI) network building and identification of hub genes

The PPI network of the 44 genes related to NDC80 complex components was constructed using the STRING database (V11.5; http://string-db.org/) to predict protein functional associations. Subsequently, the Cytoscape software (V3.9.1; http:// cytoscape. org/) was applied for the visualization of the PPI network. Then the 'betweenness' of every node was assessed by CytoNCA, a plugin of Cytoscape software. The top 10 genes, as ranked by 'betweenness', were considered the hub genes of the PPI network.

### Cell culture and transfection

Human breast cancer cell lines (BT-549 and MCF-7) were purchased from Procell Life Science & Technology Co., Ltd (Wuhan, China). BT-549 was cultured in RPMI 1640 medium supplemented with 10% FBS and insulin (10μg/mL), while MCF-7 was cultured in MEM medium supplemented with NEAA, 10% FBS and insulin (10μg/mL). Both BT-549 and MCF-7 were cultured in a humidified atmosphere containing 5% CO2 at 37℃. SiRNA oligos for NDC80 were synthesized by Genepharma (Shanghai, China). BT-549 was seeded at a density of 8×10^4^/well and MCF-7 was seeded at a density of 5×10^5^/well on coverslips in a 6-well plate for 24 hours. The cells were further transfected with siRNA using Lipofectamine 3000 (Invitrogen, USA) according to the manufacturer's instructions for 48 hours. After transfection, the cells were collected for western blotting to validate the silencing of targeted genes.

### Cell growth assays

At the end of transfection, cells were incubated with medium containing 30μM EdU for 2 hours and fixed in 4% PFA for 15 minutes. Incorporated EdU was detected using the Click-iT EdU Alexa Fluor 488 Imaging kit according to manufacturer's instructions (Thermo Fisher Scientific, USA). Images were captured using a Leica SP8 confocal microscope. The DAPI+ nuclei (representing the total cell number) and EdU+ nuclei (representing the proliferating cell number) were counted using ImageJ software. The proliferation rate was calculated using the following formula: proliferation rate = (EdU+ nuclear number / DAPI+ nuclear number) × 100%.

### Statistical analysis

Data were presented as mean ± standard deviation. The differences between 2 groups were analyzed using unpaired 2-tailed *t* test or paired *t* test. All the analyses were performed using GraphPad Prism 9 software. *P* < 0.05 was considered to be significant.

## Results

### Expression landscape of NDC80 complex components in pan-cancer

**Figure 1:**
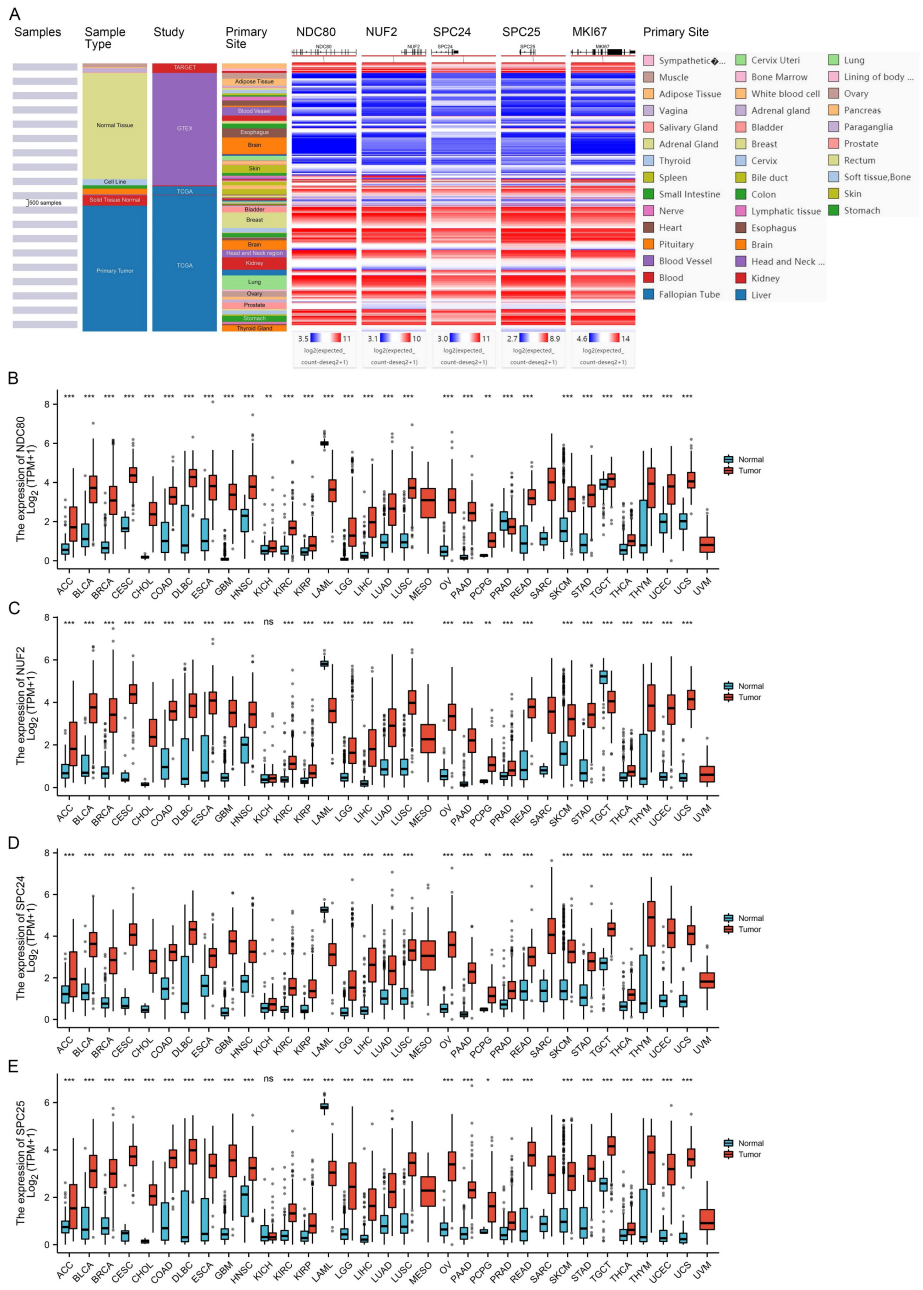
Expression of NDC80 complex components in pan-cancer. (A) Heat map of NDC80, NUF2, SPC24, SPC25, and MKI67 expression across normal and tumor tissues in the TCGA/TARGET/GTEx database obtained from UCSC Xena web-based tool. (B-E) Expression of NDC80, NUF2, SPC24 and SPC25 mRNA levels between cancer tissues and normal tissues based on TCGA and GTEx databases. (*, *p* < 0.05; **, *p* < 0.01; ***, *p* < 0.001; ns, no statistical difference).

Using the TCGA/TARGET/GTEx RNA-seq data-set which contains data from 23 cancer types and corresponding normal tissues, we analyzed the mRNA expression profile of NDC80 complex components in normal and tumor tissues. We found that similar with cell proliferation indicator - MKI67, the expression of NDC80 complex components was higher in tumor tissues than in normal tissues in the majority of cancer types (Figure 1A). Next, we corroborated the expression patterns of NDC80 complex components in 33 cancer types using the TCGA and GTEx data. We found that NDC80 complex components were significantly overexpressed in almost all TCGA cancer types, with the exception of mesothelioma (MESO), sarcoma (SARC), and uveal melanoma (UVM) due to limited availability of normal tissue data (Figure 1B-E). Interestingly, only a few cancer types demonstrated lower expression of NDC80 complex components in tumor tissues. Specifically, the levels of NDC80 were only decreased in acute myeloid leukemia (LAML) and prostate adenocarcinoma (PRAD) (Figure 1B), and NUF2 downregulation was only observed in LAML and testicular germ cell tumors (TGCT) (Figure 1C). Additionally, lower expression of SPC24 and SPC25 was only observed in LAML (Figure 1D-E).

### Correlations of NDC80 complex components expression with pathological stages in pan-cancer

**Figure 2:**
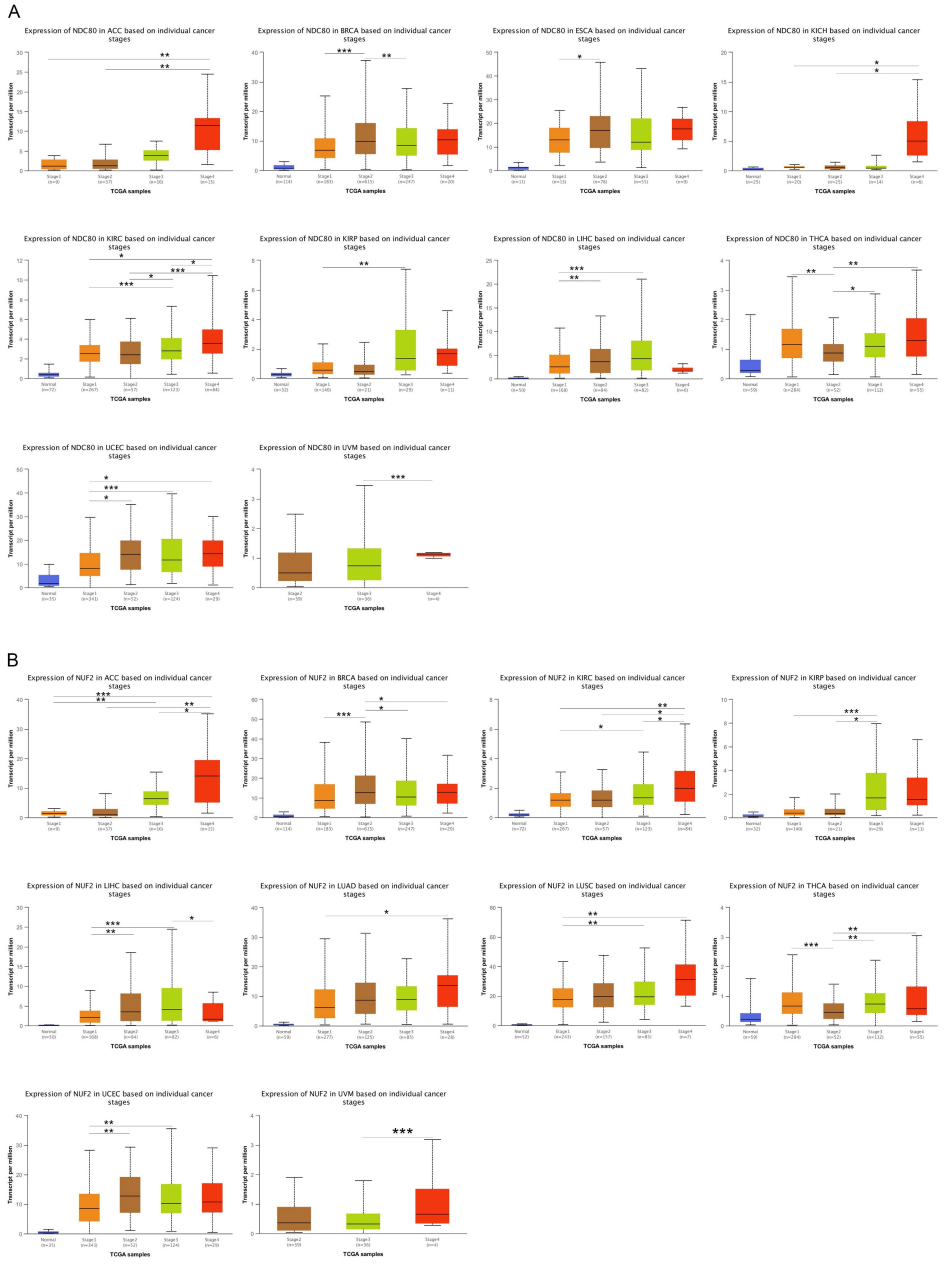

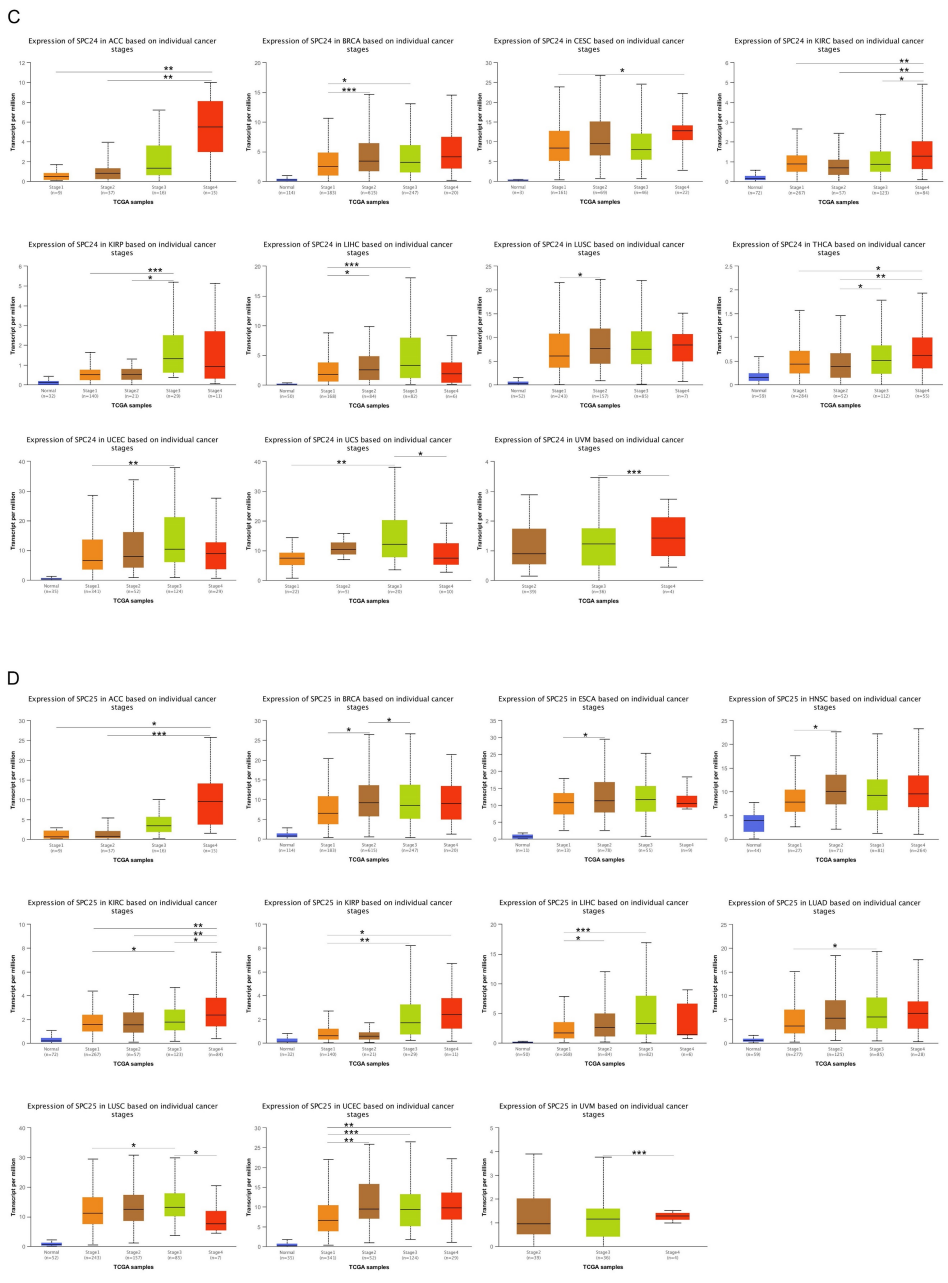
Correlation between NDC80 complex components expression and tumor stages in pan-cancer. (*, *p* < 0.05; **, *p* < 0.01; ***, *p* < 0.001).

We compared the expression levels of NDC80 complex components in different tumor stages in pan-cancer. The results indicated that the expression of NDC80 was upregulated at higher tumor stages in ACC, BRCA, ESCA, KICH, KIRC, KIRP, LIHC, THCA, UCEC, and UVM (Figure 2A). Additionally, NUF2 expression increased at higher tumor stages in ACC, BRCA, KIRC, KIRP, LIHC, LUAD, LUSC, THCA, UCEC, and UVM (Figure 2B). We observed SPC24 upregulation at higher tumor stages in ACC, BRCA, CESC, KIRC, KIRP, LIHC, LUSC, THCA, UCEC, UCS, and UVM (Figure 2C). Furthermore, the expression of SPC25 increased at higher tumor stages in ACC, BRCA, ESCA, HNSC, KIRC, KIRP, LIHC, LUAD, LUSC, UCEC, and UVM (Figure 2D).

### Prognostic value of NDC80 complex components in pan-cancer

**Figure 3:**
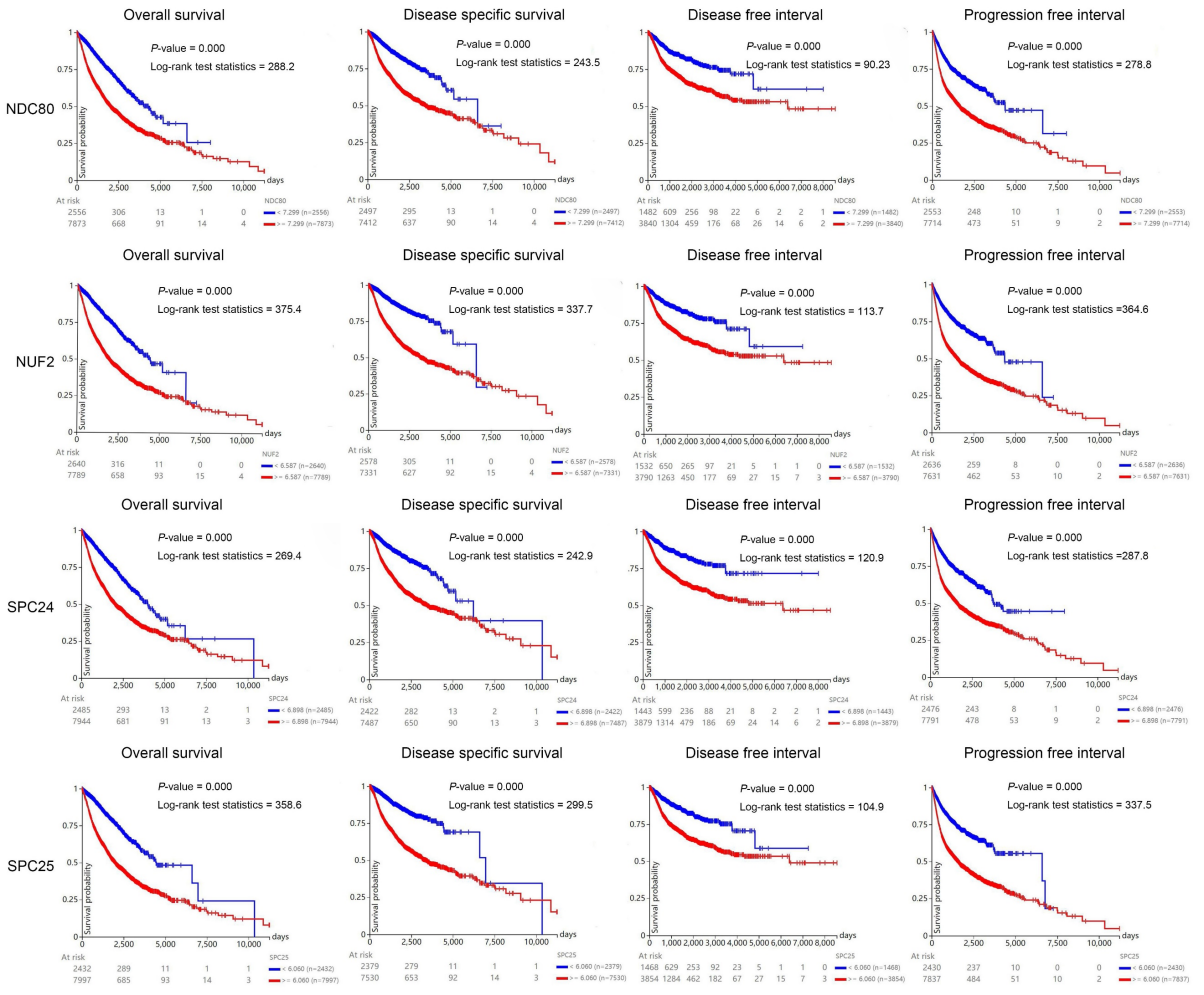
Kaplan-Meier curve of NDC80 complex components of pan-cancer samples using UCSC Xena web-based tool.

To investigate the prognostic value of NDC80 complex components in human cancers, we carried out the Kaplan-Meier analysis at the pan-cancer level. The Kaplan-Meier curve and log-rank test analysis revealed that increased NDC80, NUF2, SPC24, and SPC25 mRNA levels were all significantly associated with the overall survival (OS), disease-free survival (DFS), disease-free interval (DFI), and progression-free interval (PFI) of pan-cancer samples. The cancer patients with higher expression of NDC80, NUF2, SPC24, and SPC25 were predicted to have worse OS, DFS, DFI, and PFI, which suggested that NDC80 complex components may be potential prognostic indicator molecules in pan-cancer (Figure 3).

### Immune infiltration associated with expression levels of NDC80 complex components

**Figure 4:**
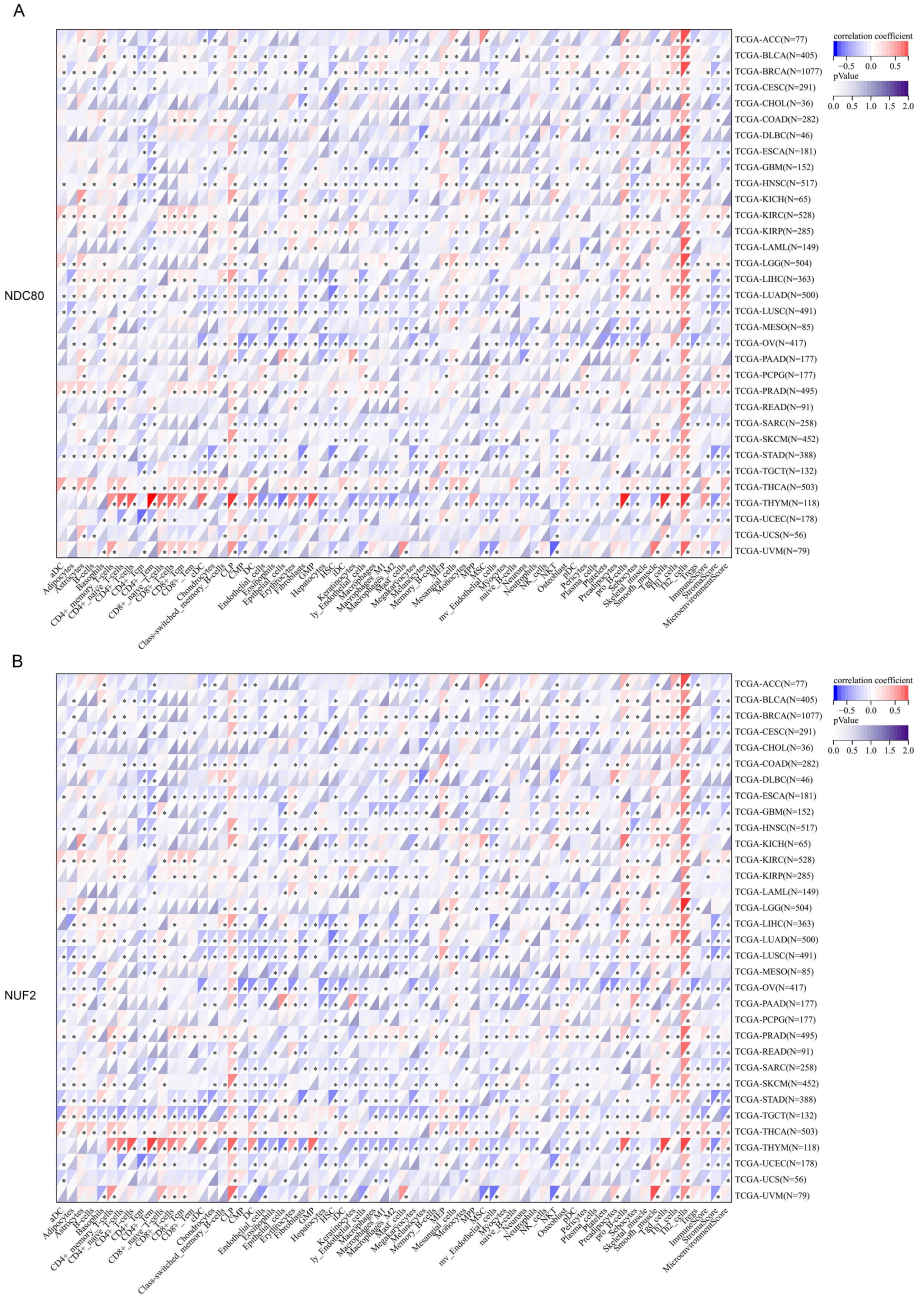

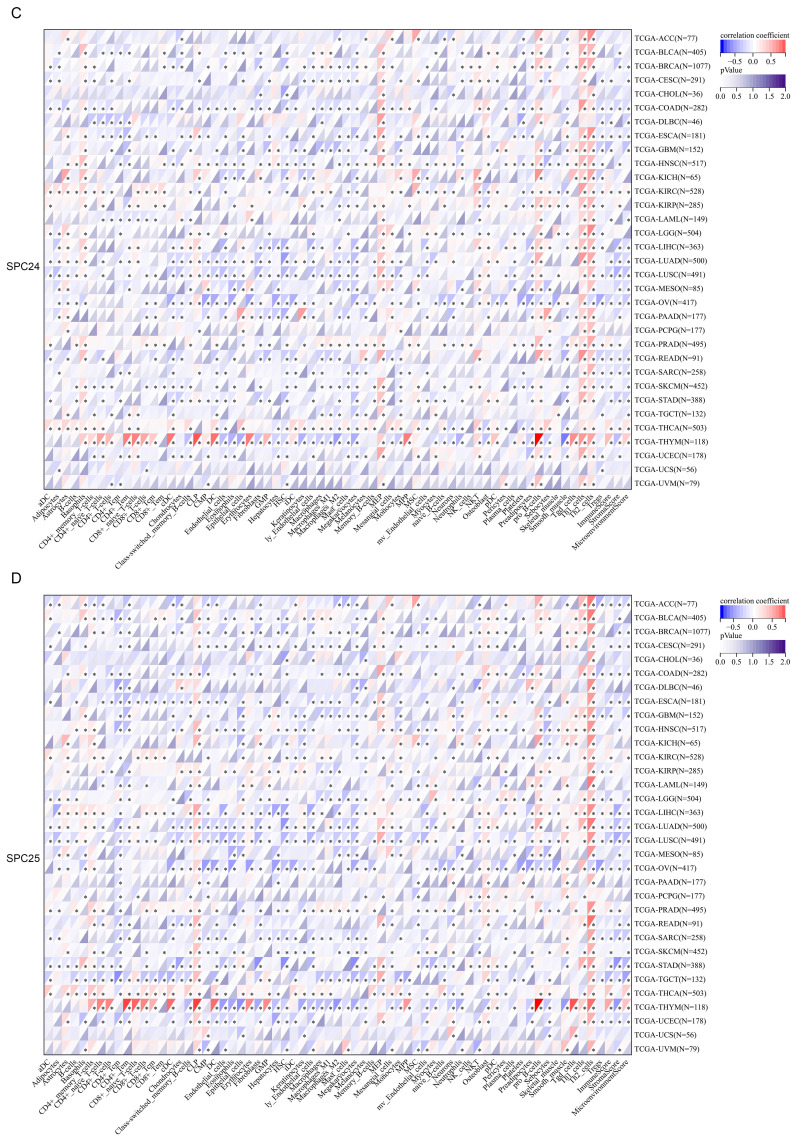
Correlation analysis between NDC80 complex components expression and immune cell infiltration in pan-cancer. (A-D) Correlation analysis between 64 immune cells infiltration and expression levels of NDC80 complex components in pan-cancer by xCell algorithm.

Next, we thoroughly analyzed the correlation between the expression of NDC80 complex components and the infiltration of 64 immune cells in pan-cancer based on the xCell algorithm. Our results revealed a strong and significant correlation between the expression of NDC80 complex components and immune cell infiltration across different TCGA cancer types. Particularly noteworthy was the finding that the expression of the NDC80 complex components showed a significant and positive correlation with Th2 cell in almost all TCGA cancer types (Figure 4A-D). Among the NDC80 complex components, SPC24 exhibited only a moderately positive correlation with Th2 cell infiltration compared with NDC80, NUF2, and SPC25. We further verified the above relationship using the TIMER2 database, which yielded consistent results (Figure S1). Taken together, these results provided strong evidence that the expression of NDC80 complex components influenced Th2 cell infiltration in pan-cancer.

### Correlation between NDC80 complex components expression and immune checkpoint genes

**Figure 5:**
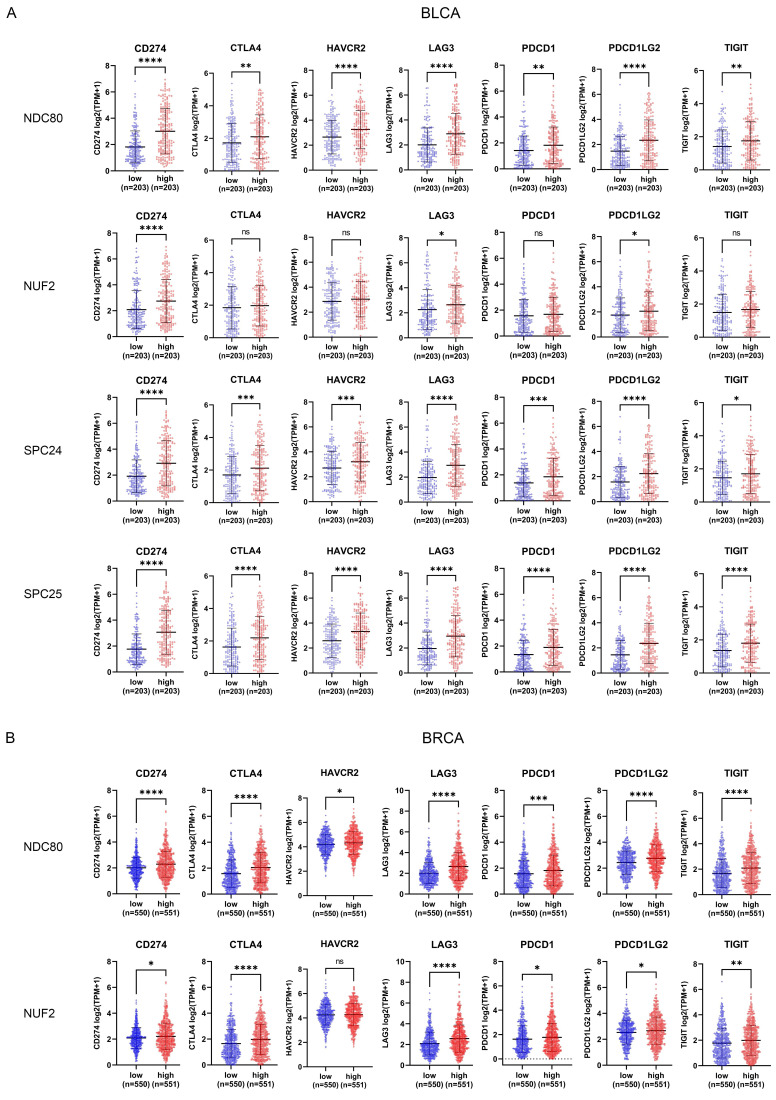

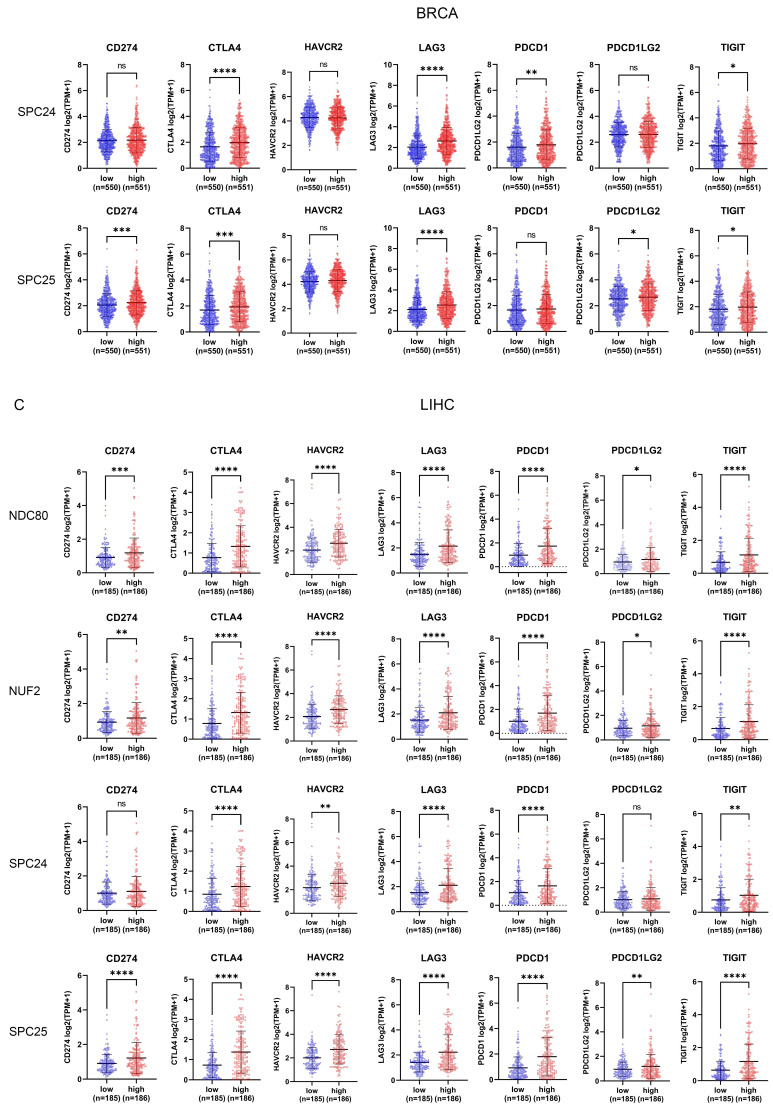
Correlation between immune checkpoint expression and NDC80 complex components expression in various cancer types. (A-C) Immune checkpoint genes expression levels between low and high NDC80 complex components expression groups in BLCA, BRCA, and LIHC. Data were analyzed by unpaired t test. Each dot represents an individual patient sample. (*, *p* < 0.05; **, *p* < 0.01; ***, *p* < 0.001; ****, *p* < 0.0001; ns, no statistical difference).

In addition to analyzing the correlation between NDC80 complex components and immune cell infiltration, we also evaluated the correlation of seven immune checkpoint genes (CD274, CTLA4, HAVCR2, LAG3, PDCD1, PDCD1LG2, and TIGIT) with NDC80 complex components expression through TCGA. The patients with BLCA, BRCA, and LIHC were classified into low and high groups according to the median expression levels of NDC80, NUF2, SPC24, and SPC25 respectively. Interestingly, BLCA patients with higher expression of NDC80 complex components exhibited increased expression of these immune checkpoint genes, although no significant differential expression was observed for CTLA4, HAVCR2, PDCD1, and TIGIT between low and high NUF2 expression groups (Figure 5A). Intriguingly, similar results were observed in BRCA and LIHC patients (Figure 5B-C), with the exception of HAVCR2, which did not show significant differential expression between low and high NUF2 expression groups in BRCA patients. Furthermore, CD274, HAVCR2, and PDCD1LG2 expression did not differ significantly between low and high SPC24 expression groups in BRCA, and HAVCR2 and PDCD1 did not show significant differential expression between low and high SPC25 expression groups in BRCA. In LIHC, only CD274 and PDCD1LG2 expression did not differ significantly between low and high SPC24 expression groups in LIHC.

We further verified the relationship between seven immune checkpoint genes and NDC80 complex components using TIMER2.0 database. The results showed a significant positive correlation between immune checkpoint genes and NDC80 complex components across various cancer types, including BRCA, BLCA, KIRC, LIHC, LUAD, and THCA (Figure S2).

### Functional states of NDC80 complex components in scRNA-seq datasets and functional enrichment of NDC80 complex components related genes in pan-caner

We investigated the functional state of NDC80 complex components in various cancer types via the CancerSEA at the single-cell resolution. The results indicated that NDC80 complex components were positively associated with the cell cycle, proliferation, DNA damage, and DNA repair in the majority of cancer types except SPC24 (Figure S3).

**Figure 6:**
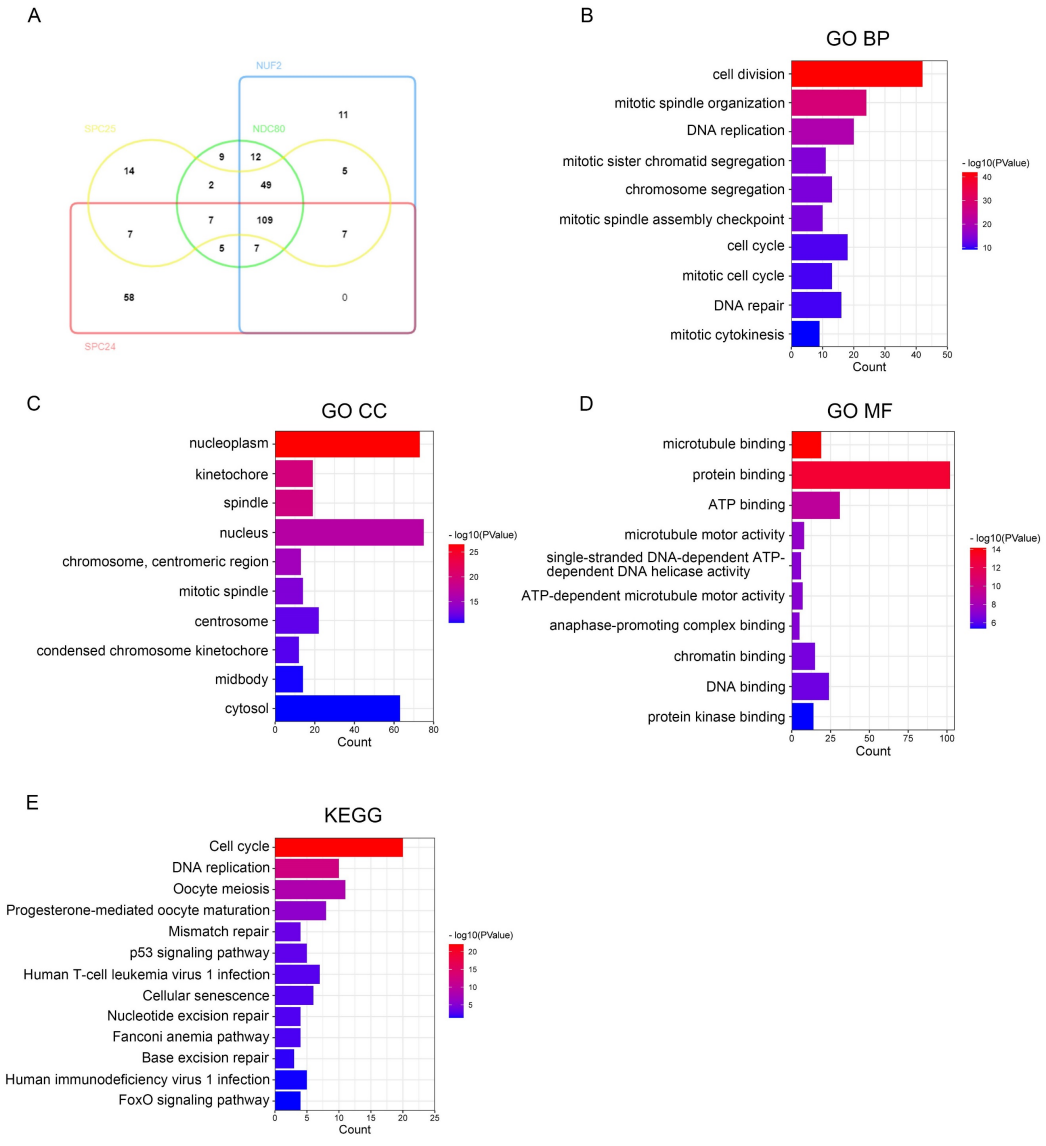

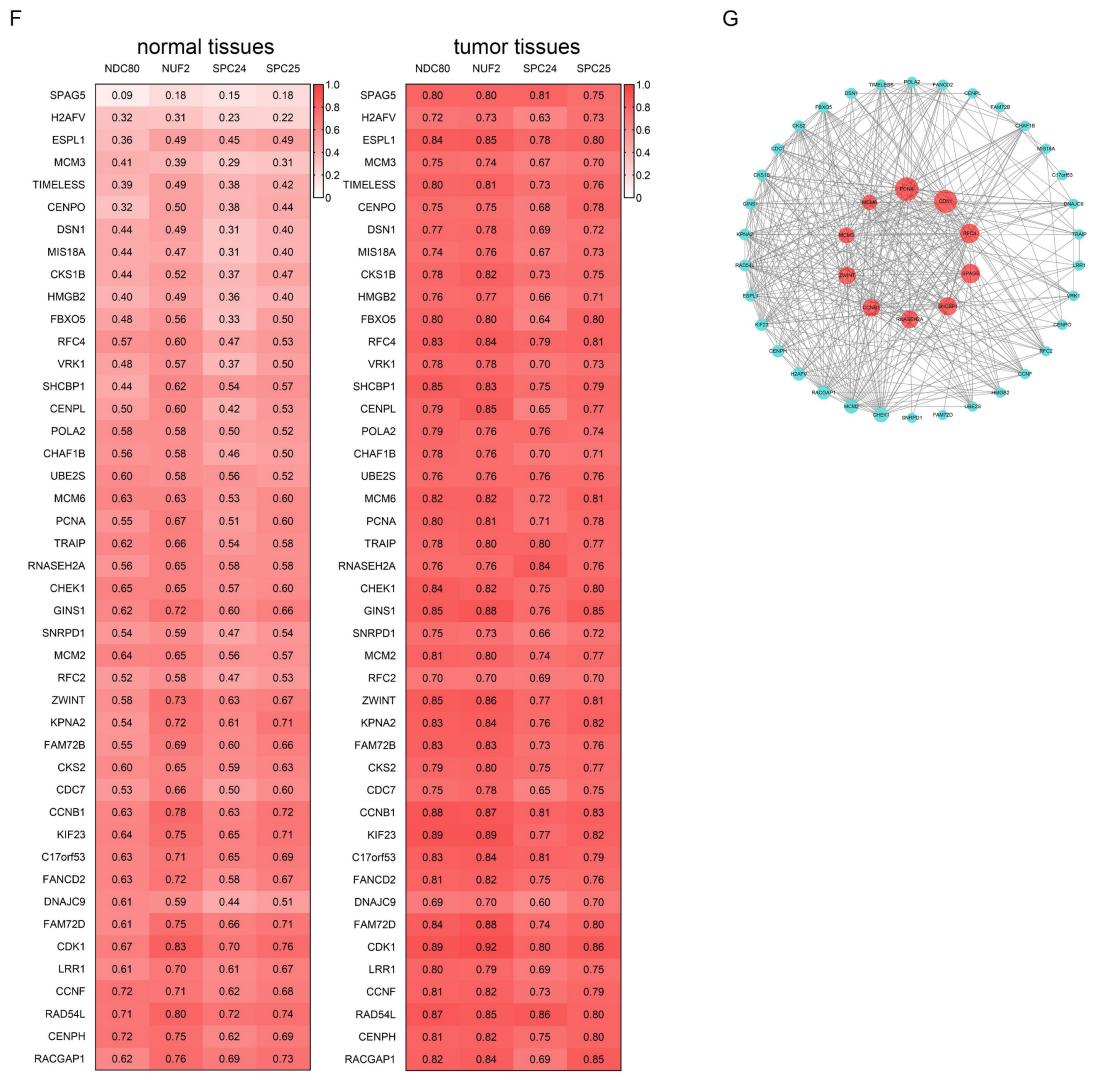
The function of NDC80 complex components related genes in pan-cancer and hub gene analysis of PPI network. (A) Venn diagram of the NDC80, NUF2, SPC24 and SPC25 top 200 related genes respectively in pan-cancer drawed by InteractiVenn tool. (B-E) GO and KEGG analysis of the 109 overlapping genes which all have high correlation with NDC80 complex components in pan-cancer by DAVID tool. (F) Correlation analysis with spearman's rank correlation coefficients of 44/109 overlapping genes and NDC80 complex components expression in TCGA normal tissues and tumor tissues by GEPIA2 database (all Spearman *p* < 0.0001). (G) PPI network of 44 overlapping genes having higher association with NDC80 complex components in the tumor context than that in normal tissues using STRING database and Cytoscape software. The size of rounds is positively associated with betweenness calculated by CytoNCA in the network, and the red rounds indicate the top 10 genes with highest betweenness.

We then used DAVID to identify the GO functional enrichment and KEGG pathway among the NDC80 complex components related genes in pan-cancer. Toward this, we performed intersection analysis on the top 200 genes having highest correlation with NDC80 complex components - NDC80, NUFS, SPC24, and SPC25 respectively at the pan-cancer level, and identified 109 overlapping genes (Figure 6A). Next, we conducted the GO and KEGG enrichment analysis on these 109 overlapping genes and found that the primary biological processes (BP) associated with them were cell division, mitotic spindle organization, DNA replication, mitotic sister chromatid segregation, chromosome segregation, mitotic spindle assembly checkpoint, cell cycle, mitotic cell cycle, DNA repair, and mitotic cytokinesis (Figure 6B). The cellular components (CC) mainly associated with the overlapping genes were nucleoplasm, kinetochore, spindle, nucleus, chromosome, centromeric region, mitotic spindle, centrosome, condensed chromosome kinetochore, midbody, and cytosol (Figure 6C). The molecular functions (MF) showed that the genes were primarily involved in microtubule binding protein binding, ATP binding, microtubule motor activity, single-stranded DNA-dependent ATP-dependent DNA helicase activity, ATP-dependent microtubule motor activity, anaphase-promoting complex binding, chromatin binding, DNA binding, and protein kinase binding (Figure 6D). The KEGG pathway enrichment showed that the overlapping genes were mainly related to Cell cycle, DNA replication, Oocyte meiosis, Progesterone-mediated oocyte maturation, Mismatch repair, p53 signaling pathway, Human T-cell leukemia virus 1 infection, Cellular senescence, Nucleotide excision repair, Fanconi anemia pathway, Base excision repair, Human immunodeficiency virus 1 infection, and FoxO signaling pathway (Figure 6E).

We further examined the association alterations between the 109 overlapping genes and the NDC80 complex components in the normal tissues and tumor tissues. To this end, we used the average Spearman *r* difference ≥ 0.1 between tumor tissues and tissues as a cut-off. Our analysis showed that the correlation between the expression of NDC80 complex components and 44 of 109 overlapping genes, including SPAG5, H2AFV, ESPL1, MCM3, TIMELESS, CENPO, DSN1, MIS18A, CKS1B, HMGB2, FBXO5, RFC4, VRK1, SHCBP1, CENPL, POLA2, CHAF1B, UBE2S, MCM6, PCNA, TRAIP, RNASEH2A, CHEK1, GINS1, SNRPD1, MCM2, RFC2, ZWINT, KPNA2, FAM72B, CKS2, CDC7, CCNB1, KIF23, C17orf53, FANCD2, DNAJC9, FAM72D, CDK1, LRR1, CCNF, RAD54L, CENPH, and RACGAP1, was significantly higher in the tumor context compared to the normal tissues (Figure 6F).

While the remaining 65 of 109 overlapping genes didn't show any significant change, including CHAF1A, C16orf59, KIF2C, POC1A, PLK4, POLE2, FEN1, NUSAP1, SGO1, WDR76, CDCA3, KIF11, MTFR2, TPX2, UBE2T, EXO1, MAD2L1, OIP5, BUB1B, TTK, PRC1, FANCI, NCAPH, CENPA, CENPF, HJURP, KIFC1, KIF15, SKA3, CDC20, CDC25C, GTSE1, TROAP, ASPM, LMNB1, CKAP2L, CDCA8, ORC6, MND1, NCAPG, KIF18B, PLK1, KIF20A, SKA1, BUB1, KIF4A, FOXM1, CDCA5, BIRC5, DLGAP5, AURKB, CDC45, EZH2, CDKN3, CCNB2, MELK, CCNA2, RAD51, CENPU, KIAA0101, ASF1B, UBE2C, MKI67, PBK, and PIMREG (data not shown). Furthermore, from the 44 overlapping genes having higher association with NDC80 complex components in the tumor context, we obtained the top 10 hub genes, including PCNA, CDK1, RFC4, SPAG5, SHCBP1, RNASEH2A, CCNB1, ZWINT, MCM3, and MCM6 (Figure 6G).

### Correlation between NDC80 complex components expression and TP53 mutation status

**Figure 7:**
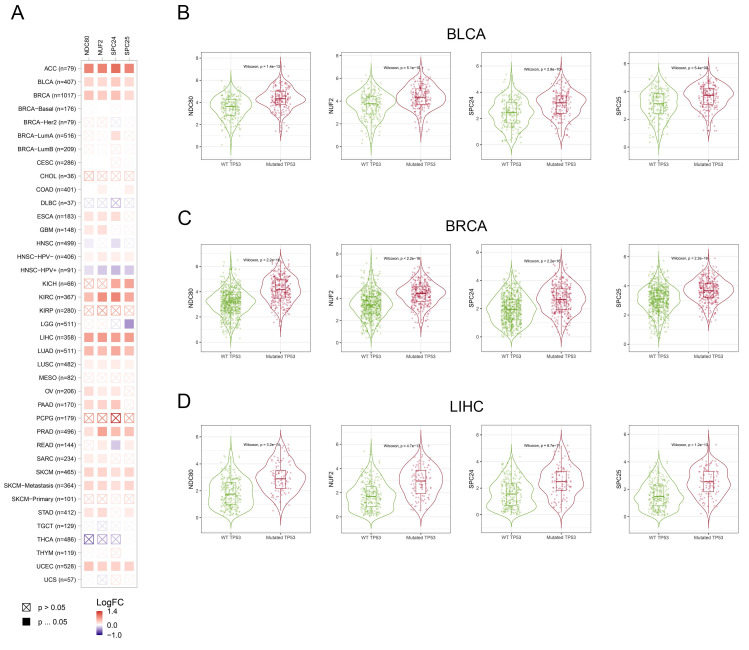
Correlation analysis between NDC80 complex components expression and TP53 mutation status in pan-cancer. (A) Correlation analysis between TP53 mutation and expression levels of NDC80 complex components in pan-cancer using TIMER2.0 database. (B-D) NDC80 complex components expression levels between WT TP53 and mutated TP53 patients in BLCA, BRCA and LIHC using TIMER2.0 database.

We assessed the correlation between the expression of NDC80 complex components and TP53 mutation in pan-cancer. The expression of NDC80 complex components showed a significant and positive correlation with TP53 mutation in different cancer types, including ACC, BLCA, BRCA, KIRC, LIHC, LUAD, PRAD, SKCM, and UCEC (Figure 7A). Additionally, BLCA, BRCA, and LIHC patients with mutated TP53 exhibited increased expression of NDC80 complex components compared to patients with wild-type (WT) TP53 (Figure 7B).

### Knockdown of NDC80 suppressed breast cancer cell growth *in vitro*

**Figure 8:**
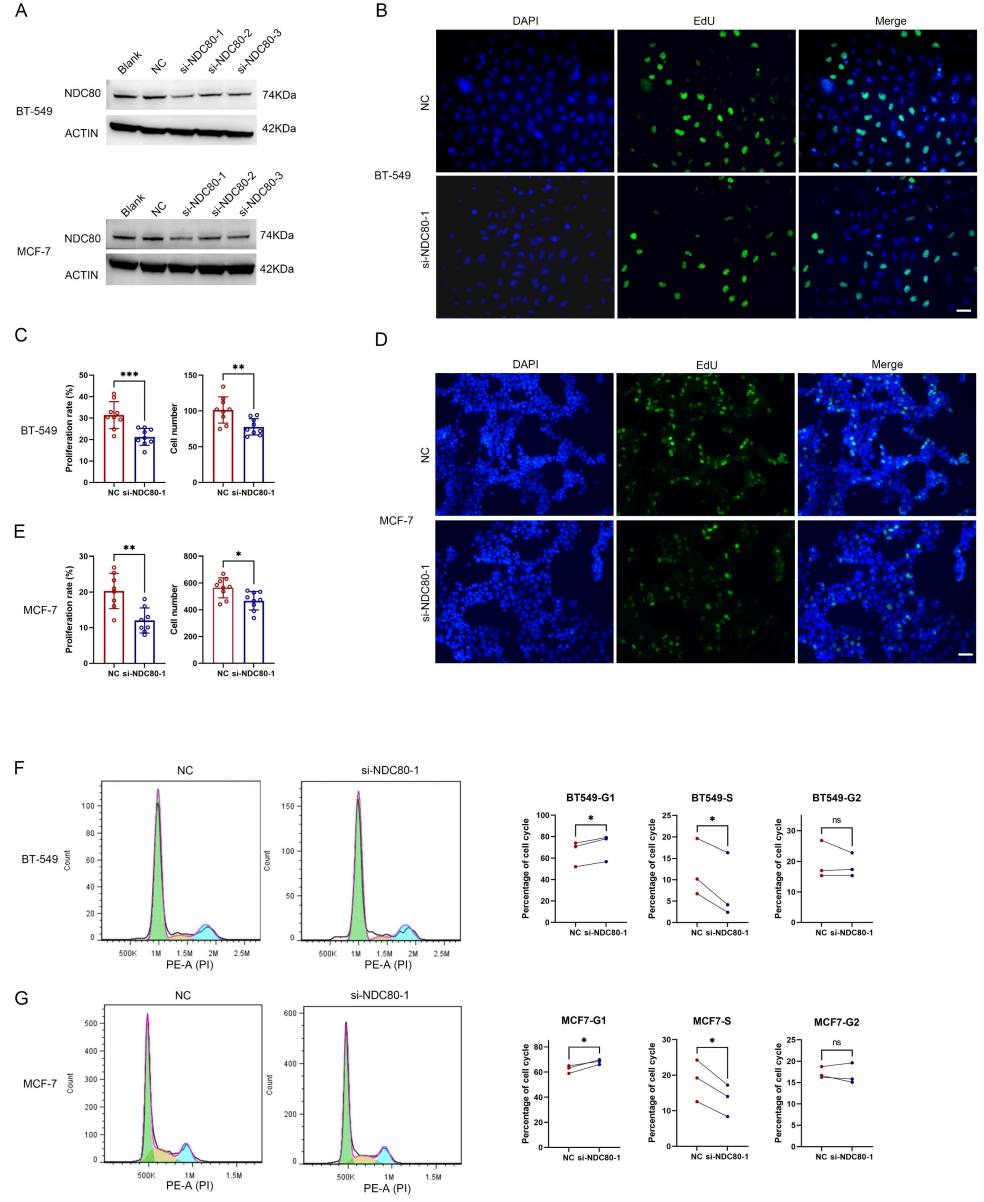
Knockdown of NDC80 suppresses breast cancer cell proliferation *in vitro*. (A) MCF-7 and BT-549 cells were untreated (Blank), or transfected with negative control (NC) or siRNAs targeting NDC80. The relative protein expression of NDC80 in MCF-7 or BT-549 cells were analyzed by western blot. (B-E) Representative microscopy images of EDU staining and cell proliferation rate and total cell numbers for MCF-7 and BT-549 cells transfected with NC or si-NDC80-1. Scale bar: 50μm. Data were analyzed by unpaired *t* test (n=9, 3 individual experiments). (F-G) Representative cell cycle data and summary of the quantitative cell cycle data analysis results for MCF-7 and BT-549 cells transfected with NC or si-NDC80-1. Data were analyzed by paired *t* test (3 individual experiments).

To further investigate the association between the NDC80 complex and cell growth in tumor cells, we utilized siRNAs targeting NDC80 to knock down its expression in two breast cancer cell lines, BT-549 and MCF-7 (Figure 8A). The most potent si-NDC80-1 was used for subsequent experiments. The EdU proliferation assay in BT-549 and MCF-7 cells transfected with negative control (NC) or si-NDC80-1 demonstrated that knockdown of NDC80 significantly suppressed the cell growth of both BT-549 and MCF-7 cells (Figure 8B-E). Furthermore, cell cycle analysis via flow cytometry revealed that knockdown of NDC80 significantly increased the percentage of cells in the G1 phase and decreased the percentage of cells in the S phase for both BT-549 and MCF-7 cells (Figure 8F-G). These findings suggested that knockdown of NDC80 inhibits breast cancer cell growth by impeding the G1-S phase transition.

## Discussion

The profiling of differentially expressed genes in pan-cancer by bioinformatics can provide valuable insights into identifying biomarkers for cancer detection and prognosis thereby improving the treatment designs for cancer patients. In this study, we explored the clinical significance of NDC80 complex components across TCGA cancer types. In accordance with previous studies, we found that the mRNA levels of NDC80 complex components were significantly upregulated in almost all TCGA cancer types suggesting an oncogenic role of NDC80 complex components in the majority of malignant tumors. Meanwhile, we found that the expression levels of NDC80 complex components were positively correlated with tumor staging in various cancer types, which verified the crucial role of NDC80 complex components in tumor progression. We also examined the prognostic value of NDC80 complex components by performing the Kaplan-Meier survival curve in pan-cancer samples and found that high expression of NDC80 complex components was a risk factor and associated with poor prognosis as indicated by OS, DSS, DFI, and PFI. Our results were consistent with previous studies on the prognosis of NDC80 complex components in certain cancer types and demonstrated that NDC80 complex components were promising prognostic biomarkers in pan-cancer.

The infiltrating immune cells are critical components in the tumor immune microenvironment (TIME) and the landscape of immune cells is a critical factor affecting tumor growth and progression, as well as patient survival^23^-^25^. However, it is unclear whether the immune infiltration in TIME is affected by NDC80 complex components. Therefore, we evaluated the correlation between the expression of NDC80 complex components and immune cell infiltration in TIME by xCell algorithm and TIMER2 database in pan-cancer and found that all NDC80 complex components were positively associated with infiltrating Th2 cell in almost all cancer types. Compelling studies have confirmed that Th2 cell supports tumor growth due to its immunosuppressive effect in TIME, and increased levels of Th2 cell portend worse prognosis in various cancer types^25^. Our results suggested upregulated NDC80 complex components in the tumor context may promote tumor cancer progression and influence patient survival by interacting with Th2 cell infiltration.

Checkpoint molecules play a crucial role in tightly control the immune system by regulating the T cell activation. Unfortunately, tumor cells can exploit this mechanism by upregulating inhibitory immune checkpoints, ultimately dampens the activation of T cells and leads to inhibition of anti-tumor immune response in TIME. An interesting association was found between the components of the NDC80 complex and seven inhibitory immune checkpoints (CD274 (PD-L1), CTLA-4, HAVCR2 (TIM3), PDCD1 (PD-1), PDCD1LG2 (PD-L2), TIGIT), which are the most frequent targets in current immunotherapy^26^. The results indicated that patients with high expression of NDC80 complex components had higher expression levels of immune checkpoints in certain cancer types, such as BLCA, BRCA, and LIHC. These findings suggested that NDC80 complex components may play a crucial role in regulating the expression of immune checkpoints and ultimately impact the efficacy of immunotherapy in cancer patients.

To further investigate the potential function of NDC80 complex components in tumor context, we preformed the functional state of NDC80 complex components in various cancer types through CancerSEA at the single-cell level. The results from CancerSEA indicated that the expression of NDC80 complex components was mainly positively correlated with the cell cycle, proliferation, DNA damage, and DNA repair. Further, functional enrichment analysis of NDC80 complex components related genes in the tumor context was performed. The GO analysis identified several major pathways, including cell division, mitotic spindle organization, DNA replication, mitotic sister chromatid segregation, chromosome segregation, mitotic spindle assembly checkpoint, cell cycle, mitotic cell cycle, DNA repair, and mitotic cytokinesis were the major pathways. Similarly, the KEGG pathway analysis revealed cell cycle and DNA replication as the major pathways. Taken together, these results suggested that NDC80 complex components play essential roles in promoting cell growth through cell division and cell cycle, and they were also engaged in the DNA damage and repair.

Next, we investigated the association alterations of NDC80 complex components to 109 overlapping genes between normal and tumor tissues, and we observed that 44 of 109 overlapping genes had a higher association with NDC80 complex components in the tumor context compared with normal tissues. It is worth pointing out that the majority of the 44 genes play known oncogenic roles in cancer, especially SPAG5^27^, FBOX5^28^, RFC4^29^, VRK1^30^, MCM6^31^, PCNA^32^, KPNA2^33^, CDC7^34^, CCNB1^35^, CDK1^35^, and CCNF^36^. These oncogenes are overexpressed in multiple human cancer types and are associated with poor prognosis. Moreover, these genes are also implicated in cell cycle progression and cell proliferation. Together, our results indicated that these genes having higher association with NDC80 complex components in tumor tissues compared to normal tissues were functional partners associated with the oncogenic roles involved in cell growth of NDC80 complex components in the tumor context.

It is widely acknowledged that TP53 normally acts as a tumor suppressor gene because wild-type TP53 induces growth arrest or apoptosis. However, mutation abolishes these tumor-suppressive functions and, in some instances, confers novel biological properties to TP53. Mutated TP53 has been shown to transactivate numerous genes involved in tumor initiation and progression. For example, mutated TP53 has been demonstrated to transactivate PCNA, CCNA, CCNB, CDK1, and CDC25C, all of which can promote tumor cell growth^37^. Given that the NDC80 complex is highly correlated with cell proliferation and that tumor patients with mutated TP53 exhibit increased expression of NDC80 complex components, it is highly likely that mutated TP53 transactivates the expression of NDC80 complex components and promotes cell proliferation. However, the possibility cannot be excluded that the elevated expression of NDC80 complex components may drive the mutation of TP53.

There are limitations to our study. For instance, the comprehensive and systematic analysis of NDC80 complex components was derived from selected databases. Therefore, further studies are required to validate the roles of NDC80 complex components in Th2 cell infiltration, checkpoint genes, and TP53 mutation, and to unravel the exact mechanisms by which the NDC80 complex participates in these functions. This could provide functional evidence for our analysis. Our study also lacks experimental evidence on whether the influence of NDC80 complex components on tumor cell growth is universal in pan-cancer and the specific molecular mechanisms by which NDC80 complex components regulate tumor cell growth.

## Conclusion

In conclusion, our study demonstrated that the NDC80 complex components were overexpressed in tumors compared with normal tissues, and high expression of the NDC80 complex components was associated with poor prognosis in pan-cancer. Interestingly, our findings suggested that NDC80 complex components may play pivotal roles in Th2 cell infiltration, immune checkpoint regulation and TP53 mutation for the first time. Collectively, our results suggested that NDC80 complex components could serve as biomarkers for cancer detection and prognosis, as well as serve as cancer-promoting genes involved in Th2 cell infiltration, immune checkpoint, TP53 mutation and cell growth.

## Supplementary Material

Supplementary figures.

## Abbreviations

LUAD: lung adenocarcinoma
PAAD: pancreatic adenocarcinoma
COAD: colon adenocarcinoma
LIHC: liver hepatocellular carcinoma
BC: breast cancer
THCA: thyroid adenocarcinoma
PRAD: prostate adenocarcinoma
LUSC: lung squamous cell adenocarcinoma
TCGA: the cancer genome atlas
GTEX: Genotype-Tissue Expression
OS: overall survival
DSS: disease-specific survival
DFI: disease-free interval
PFI: progression-free interval
GO: Gene Ontology
KEGG: Kyoto Encyclopedia of Genes and Genomes
MESO: mesothelioma
SARC: sarcoma
UVM: uveal melanoma
LAML: acute myeloid leukemia
PRAD: prostate adenocarcinoma
TGCT: testicular germ cell tumors
BP: biological processes
CC: cellular components
MF: molecular functions
TIME: tumor immune microenvironment
