# Supplementary material for: Implications of the NDC80 complex on the tumor immune microenvironment and cell growth in pan-cancer
Source: J Cancer. 2024 Oct 14;15(19):6364–82. doi: 10.7150/jca.96070 (PMC11540516; doi:10.7150/jca.96070)
Supplement: Supplementary file 1 — Supplementary figures. [file jcav15p6364s1.pdf]

sFigure 1

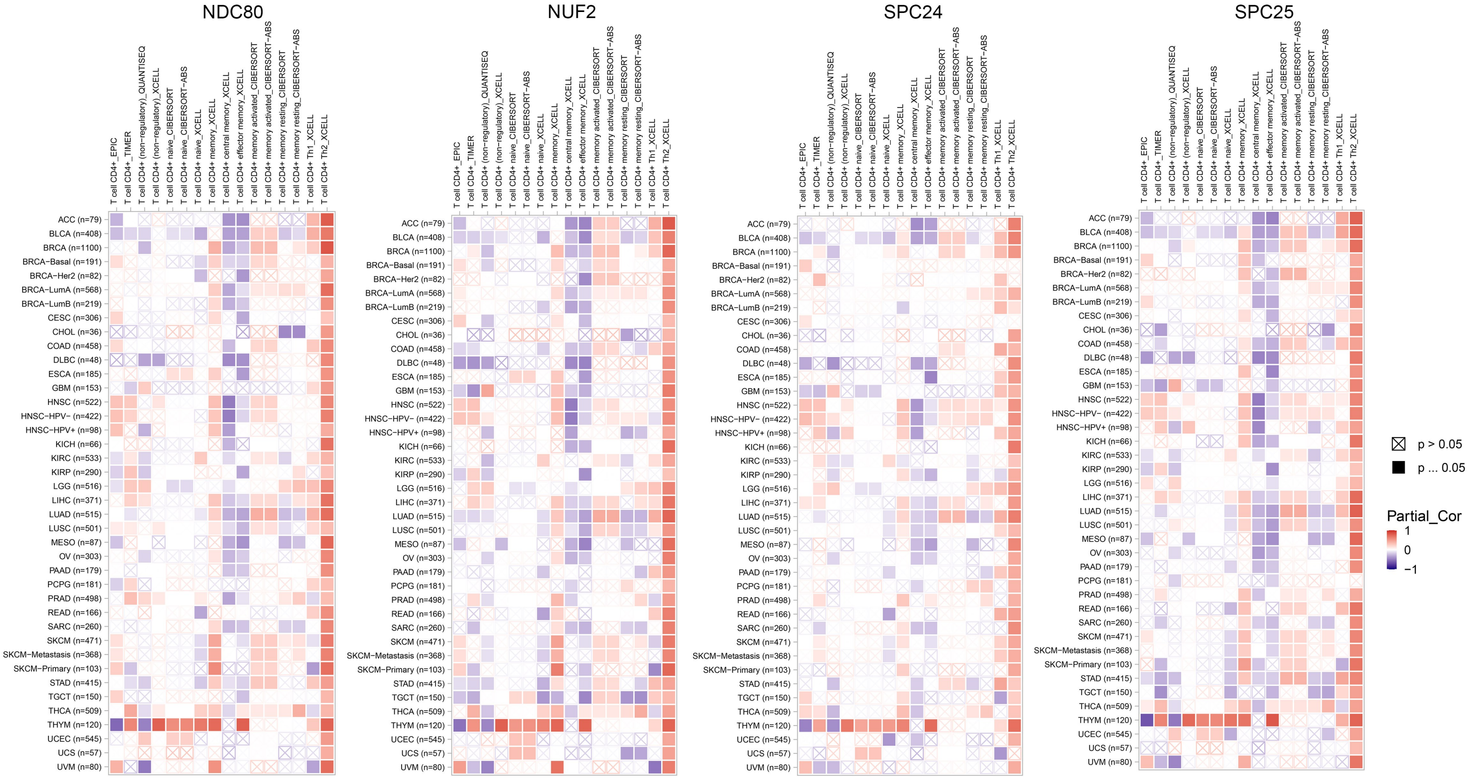

sFigure 1. Correlation analysis between NDC80 complex components expression and CD4+ T cell subsets infiltration in pan-cancer by TIMER2.0 database.

sFigure2

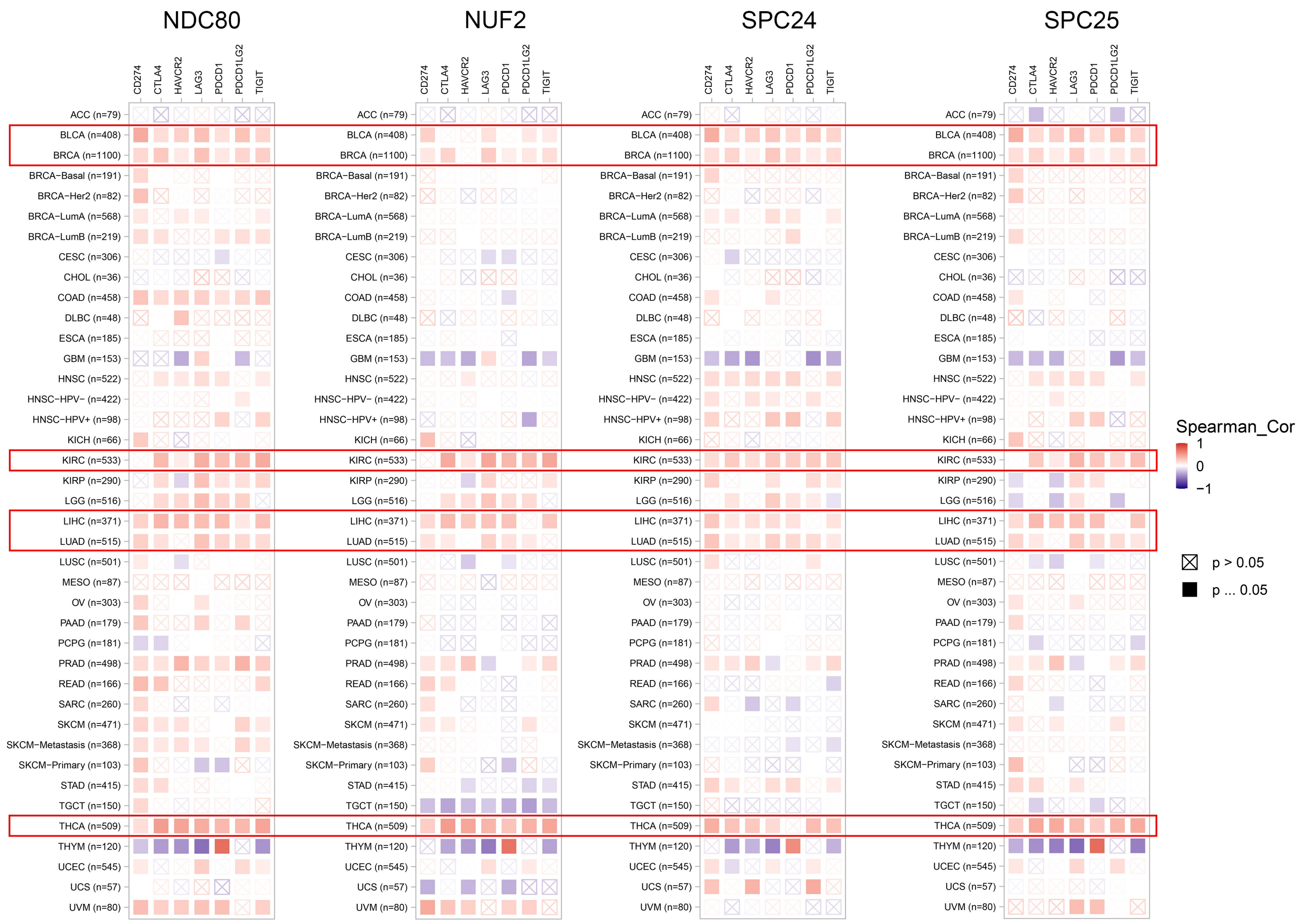

sFigure 2. Correlation analysis between NDC80 complex components and immune checkpoint in pan-cancer by TIMER2.0 database.

sFigure 3

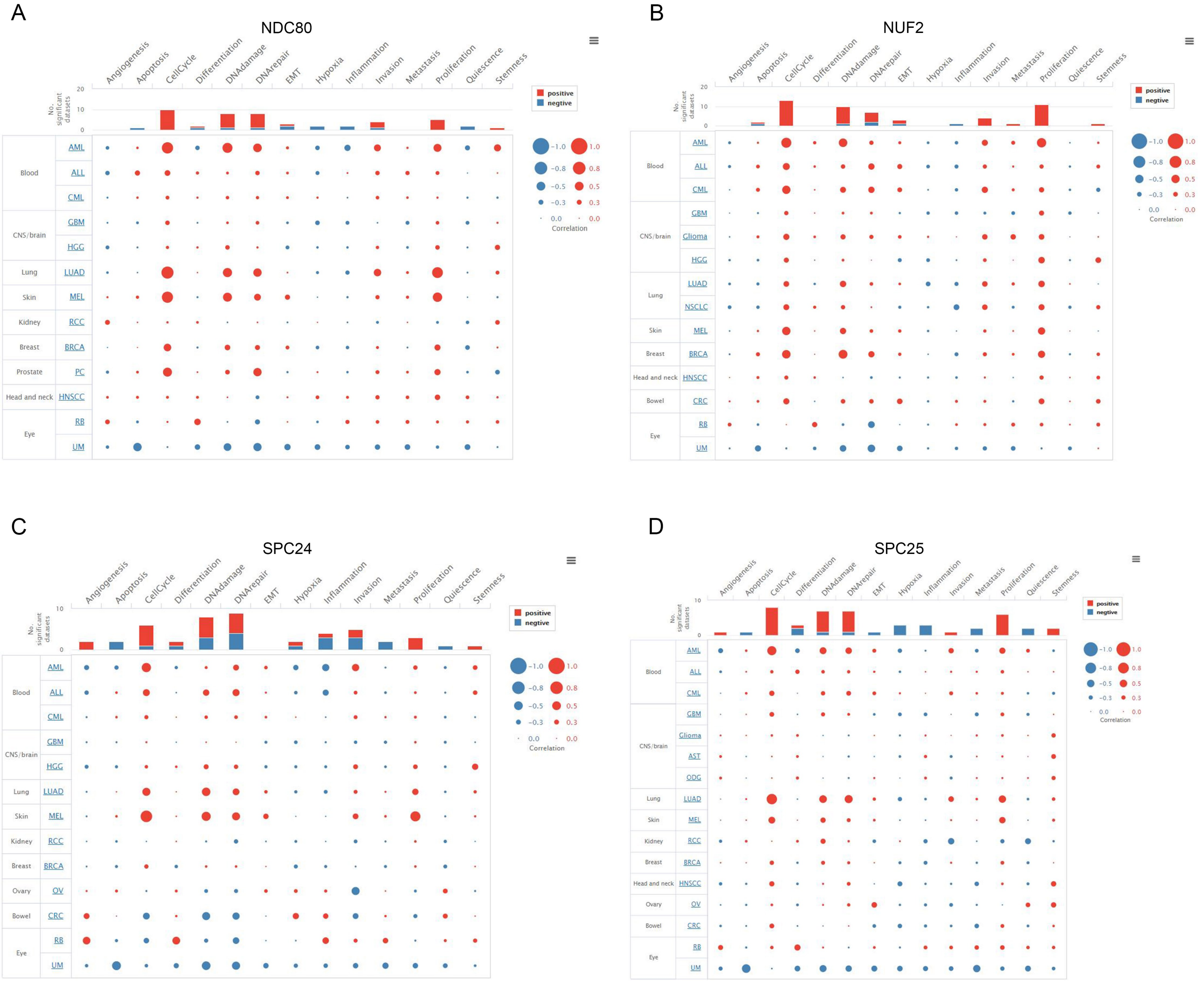

sFigure 3. The correlation of NDC80 complex components with functional states in cancers by CancerSEA. (A-D) The interactive bubble charts present correlation of NDC80 complex components with functional states in pan-cancer.
